# Supplementary figures and images for: The clinical significance of collagen family gene expression in esophageal squamous cell carcinoma
Source: PeerJ. 2019 Oct 4;7:e7705. doi: 10.7717/peerj.7705 (PMC6779144; doi:10.7717/peerj.7705)

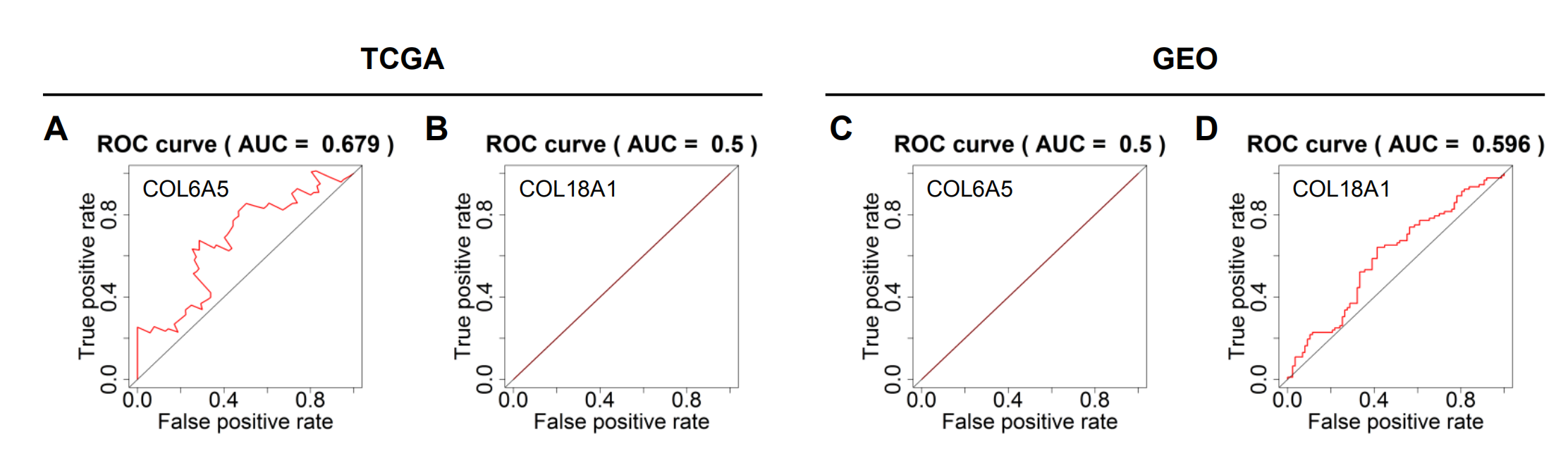

Supplement: Supplemental Information 9 — (A) and (B) ROC curves of COL6A5 in the two datasets. (C) and (D) ROC curves of COL18A1 in the two datasets. [file peerj-07-7705-s009.png]

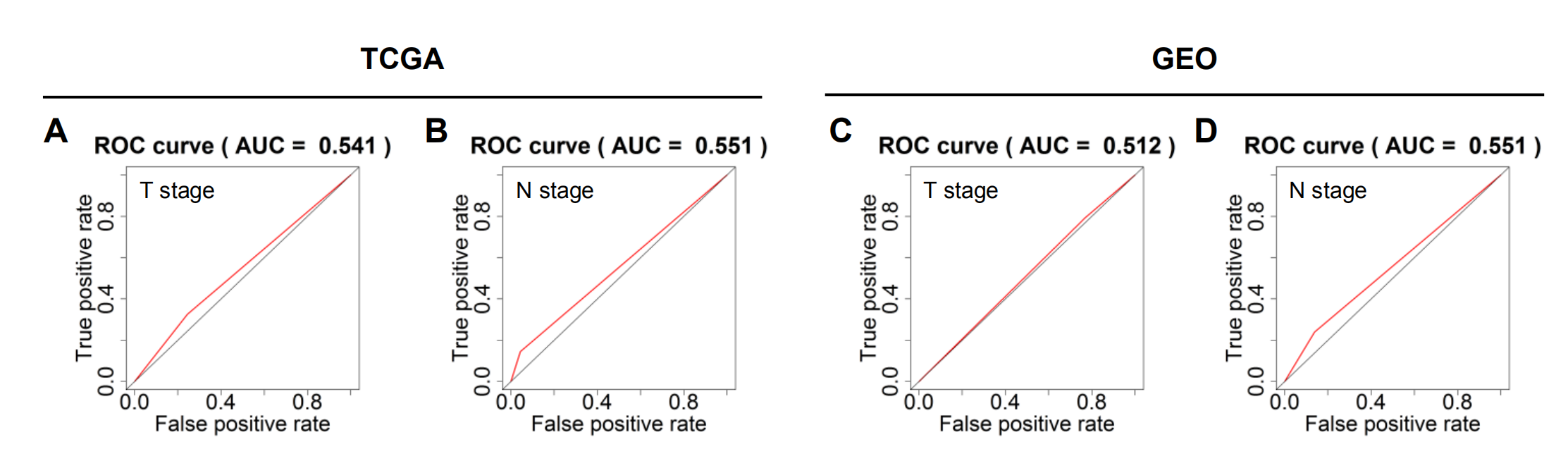

Supplement: Supplemental Information 10 — (A) and (B) ROC curves of T-stage in the two datasets. (C) and (D) ROC curves of N-stage in the two datasets. [file peerj-07-7705-s010.png]

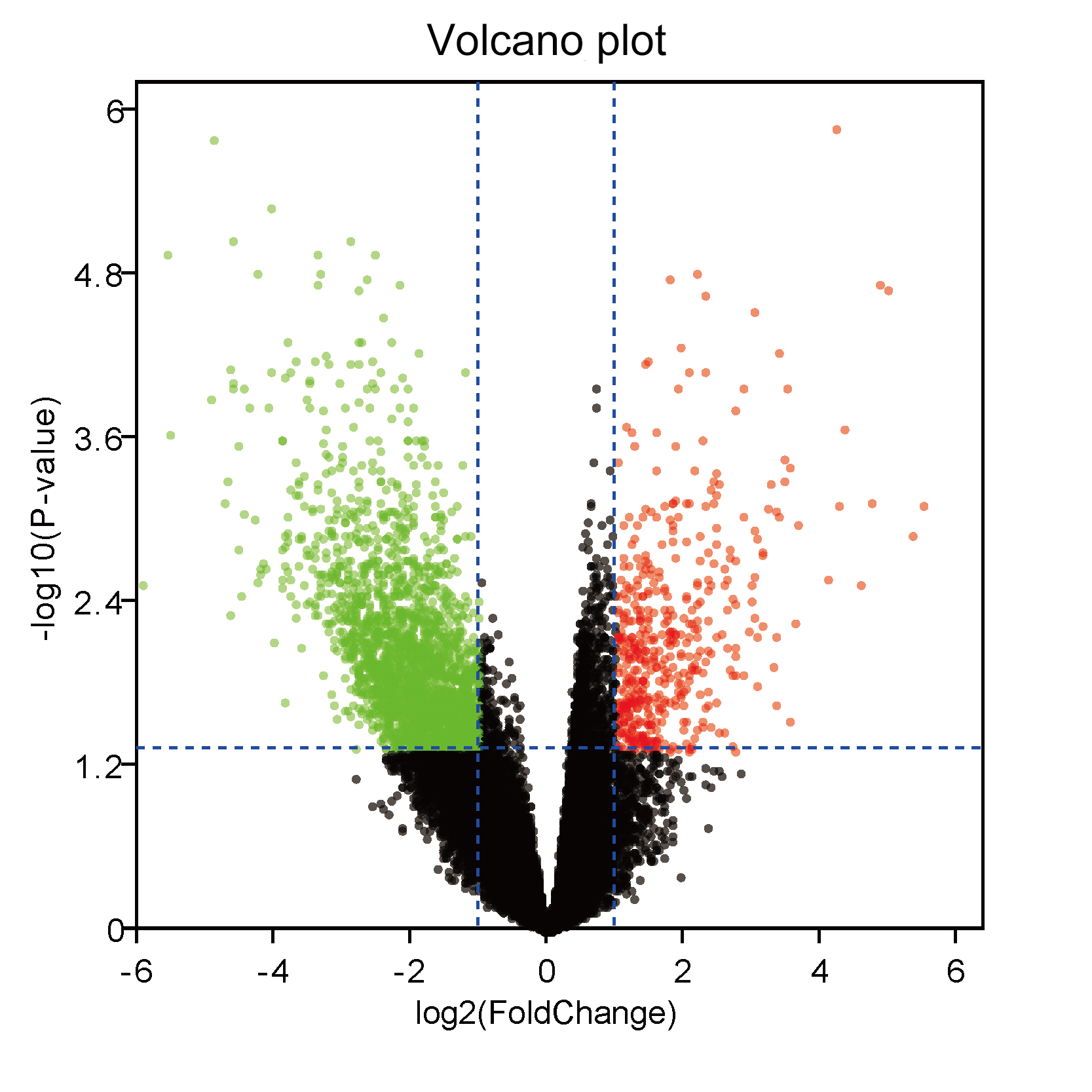

Supplement: Supplemental Information 11 — Red and green dots indicate up- and down-regulated genes, respectively. [file peerj-07-7705-s011.png]

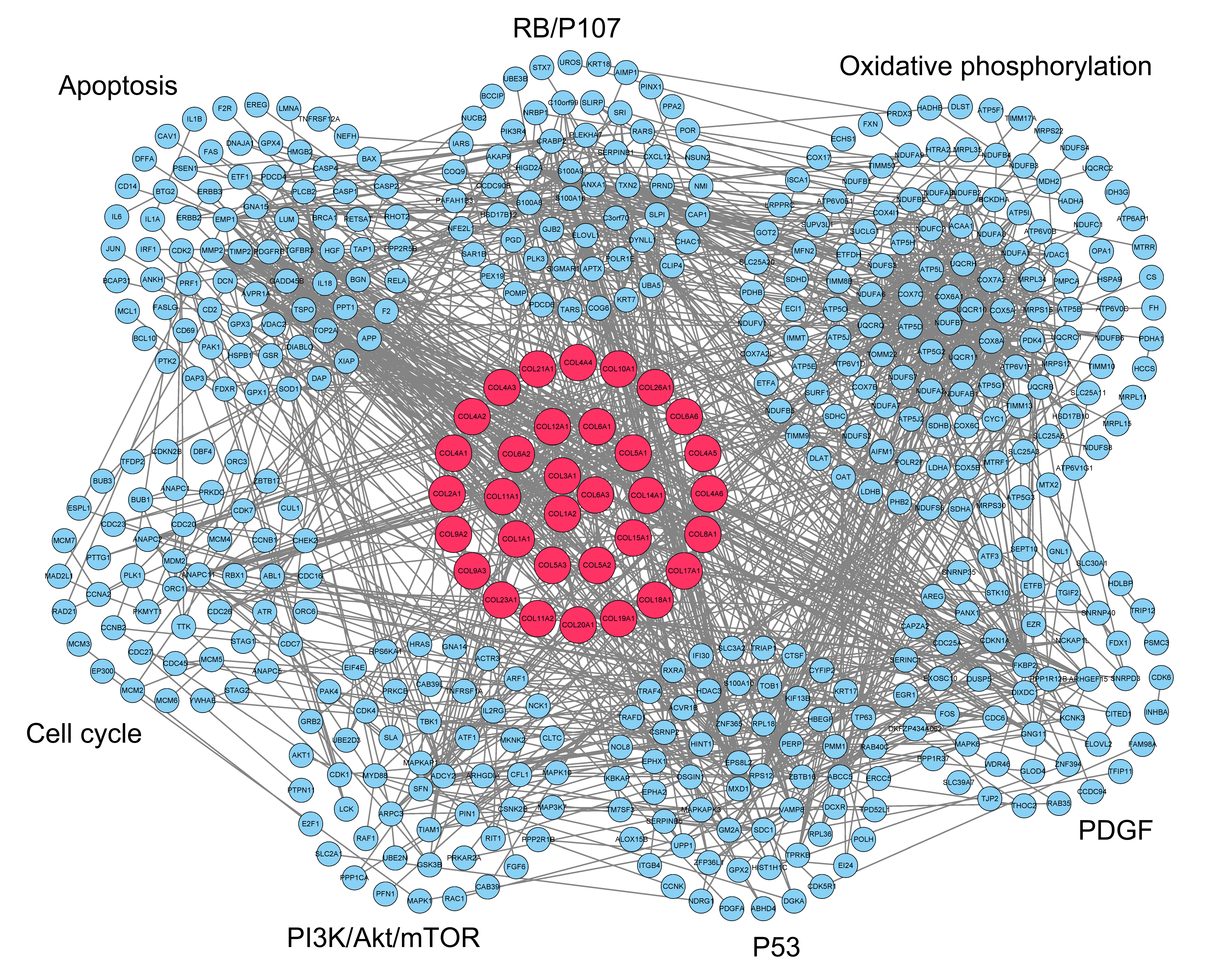

Supplement: Supplemental Information 12 — Red nodes are the collagen family genes closely correlated with those gene sets. A big blue circle represented a gene set and the blue nodes were genes included in each gene set. [file peerj-07-7705-s012.png]
